# The relationship between psychological distress and weight maintenance in weight cycling: mediating role of eating behavior

**DOI:** 10.1186/s12889-024-18349-5

**Published:** 2024-03-26

**Authors:** Wanyang Li, Dan Wang, Hongyu Chen, Yan Liu, Shuyao Dong, Mingyao Sun, Wei Chen

**Affiliations:** 1 Department of Clinical Nutrition, Chinese Academy of Medical Sciences - Peking Union Medical College, Peking Union Medical College Hospital, No. 1 Shuaifuyuan, Dongcheng District, 100730 Beijing, China; 2 Center for Behavioral Health & School of Social Development and Public Policy, Beijing Normal University, 19 Xinjiekou Wai Street, 100875 Beijing, China; 3 School of Nursing, Chinese Academy of Medical Sciences & Peking Union Medical College, Peking Union Medical College Hospital, Beijing, China; 4 Shandong Institute of Prevention and Control for Endemic Disease, 250014 Jinan, China

**Keywords:** Weight cycling, Weight loss outcomes, Psychological distress, Eating behavior, Mediating factors

## Abstract

**Background:**

Obesity is a global public health concern. The goal of this study was to see if eating habits could mediate the relationship between psychological distress and weight maintenance in a population with a history of weight cycling.

**Methods:**

A 3-month outpatient intervention consisting of a diet and exercise program was provided to 153 participants. Psychological distress, appetite, and behavior were assessed at the beginning and end of the study. Anthropometric measurements were taken at baseline and six months.

**Results:**

After the structural equation model was developed, it was discovered that the psychological status of people with obesity and weight cycling histories correlated with the weight loss outcome effect (three and six months). This effect was mediated by factors related to eating behavior. Associative psychological factors had a direct effect on eating behavior (three months: β = 0.181, 95% CI: 0.055–0.310; six months: β = 0.182, 95% CI: 0.039–0.332) and appetite had a direct effect on eating behavior (three months: β = 0.600, 95% CI: 0.514–0.717; six months: β = 0.581, 95% CI: 0.457–0.713), both of which were significant (*p* < 0.01). At three months, psychological distress has a more substantial positive impact on weight change, with eating behavior acting as a partial mediator. At six months, there was no support for appetite’s moderating role in eating behavior.

**Conclusions:**

The findings suggest that psychological interventions should be strengthened to improve weight loss effectiveness, particularly in participants with a history of weight cycling, making weight loss more complicated and prone to rebound.

**Clinical trial registration:**

The study has been registered in Clinical Trials (NCT05311462).

## Introduction

The obesity epidemic is a global public health issue. In 2016, approximately 1.9 billion adults were people with overweight, and over 650 million adults were people with obesity [1]. According to the World Health Organization (WHO), approximately 60% of European adults were people with overweight or obesity as of 2022 [2]. Severe obesity increases the risk of various diseases, including adverse cardiovascular events, non-alcoholic fatty liver disease, and cancer, thereby endangering the lives of the population with obesity [3–5]. However, research indicates that even when using the same weight loss strategy, there is significant individual variation in the degree of weight loss among people with obesity. This often results in a cycle of losing weight and regaining it, complicating long-term weight maintenance [6]. Weight maintenance is defined as the intentional loss of weight and subsequent maintenance of that weight for at least six months [7]. Weight cycling is a cyclical cycle of weight loss (≥ 5 kg) and weight regain, affecting 20–30% of adults [7, 8]. Several studies have found that weight cycling increases weight, the metabolic burden and chances of developing chronic disease of people with obesity [9, 10].

Most current medical weight loss interventions combine lifestyle changes, such as diet and exercise, with medication and surgery being recommended for those with severe obesity and co-morbidities [10]. Energy restriction and changes in macronutrient distribution are common dietary interventions, and exercise interventions alternate between aerobic and resistance exercise training. However, adhering to these exercise and healthy eating plans is challenging for most participants, especially those who have experienced weight cycling. After multiple weight regains, physiologically in terms of body composition changes and metabolic alterations and psychologically in terms of internal drive [11].

Depression, anxiety, and other forms of distress are referred to as psychological distress [12, 13]. Previous studies have established a strong association between psychological issues such as anxiety and depression with weight cycling or weight regain [14, 15]. Large-sample statistical analyses have revealed that populations with weight cycling were more likely to experience depressive symptoms than non-weight cyclers and that there was a link between frequent weight cycling and depression [16]. In addition, participants with bipolar disorder and a history of weight cycling, particularly those who often regained lost weight or struggled to sustain their weight loss, experienced more frequent relapses of symptoms such as mania and depression [17].. A subsequent study discovered that adults with baseline anxiety and depression exhibited lower levels of weight maintenance over two years, were more likely to gain weight, and that depression severity had a dose-response effect with significant weight gain [18–20]. In contrast, some remission of major depression and dysthymia was linked to improved weight control and loss. It indicated a clear relationship between psychiatric and psychological distress, weight cycling, and weight maintenance. Mental and psychological distress have been identified as essential factors in the development of certain diseases. Psychological stress has an impact on irritable bowel syndrome. Severe mental illness and cardiovascular disease have some root causes in common, and those who have a mental illness are more likely to develop cardiovascular disease [21, 22]. Currently, studies on obesity-related diseases suggests that mental stress may influence changes in eating behavior and metabolic disorders among diseases associated with obesity. According to an increasing number of studies, independent mental diseases are closely related to the occurrence of metabolic diseases [23]. We hypothesize that reducing psychological distress in people with obesity and a history of weight cycling will lead to better weight-loss outcomes.

Eating behavior can also be influenced by psychological distress. Previous cross-sectional studies have discovered a link between psychological distress and emotional eating, binge eating, and external eating. Emotional eating behaviors, in general, are mediators of the effect of psychological distress on health-related quality of life [24]. Psychological distress regulation is strongly linked to problematic eating behavior in adolescents [25]. The indirect effect of psychological distress on emotional eating via emotional dysregulation was found to be significant in mediation analyses [26]. Furthermore, 25% of adults eligible for weight loss surgery exhibit food addiction and binge eating behavior, which is linked to a high prevalence of psychological distress, including depression and anxiety disorders [27]. It is important to note that eating behavior has been linked to both psychological distress and weight loss levels. Previous research has found that changes in eating habits for weight loss are associated with better outcomes [28]. The presence and severity of eating behaviors may play a role in mediating the relationship between psychological distress and weight loss levels. However, most prior studies have primarily focused on individual factors like depression, anxiety, attitudes towards healthy eating, or exercise habits, only examining their isolated impact on weight maintenance [29].

There have been few studies that look at the link between psychological distress, eating habits, and weight loss maintenance. We proposed a hypothesis that explores a sequential pathway involving psychological status, eating behavior, and weight control. This hypothesis is based on theoretical assumptions derived from existing research in the field, suggesting a complex interplay between these factors in individuals experiencing weight cycling. Our study aims to empirically test this pathway to better understand the mechanisms underlying successful weight management. This sequence is speculated to influence the effectiveness of weight loss in populations experiencing weight cycling. We used a cohort of weight loss clinic participants in this study. This study looked at the relationship between psychological distress, appetite, eating behavior, and weight loss outcomes in outpatients who had a history of weight cycling. It also examined whether the mediation effect of eating behavior between psychological status and body mass index (BMI) change found in previous studies can be replicated and whether this mediation effect is dependent on appetite. Using a structural equation model (SEM) for path analysis, we assessed the mediation and moderated mediation between psychological status, eating behavior, and weight loss. To the best of our knowledge, this is the first study to investigate the relationship between psychological distress and weight control in a cohort of participants with obesity and a history of weight cycling, as well as to mediate the role of eating behavior. This evidence will inform future improvements in psychological interventions for participants with a history of obesity and weight cycling (people experiencing weight loss difficulties), as well as provide a theoretical foundation for advancements in multi-disciplinary team (MDT) weight loss intervention strategies aimed at promoting physical and mental health.

## Method

### Participants

The Population with Weight Cycle Obesity Study at Peking Union Medical College Hospital was used in this investigator-initiated cohort study. The goal was to investigate the mechanisms and interventions that explain the differences in weight maintenance outcomes in this population. The study included outpatients recruited in the Clinical Nutrition Department of Peking Union Medical College Hospital in April 2022. Participants had purposefully lost and regained weight before attending the clinic. Weight loss prior to the visit occurred on its own and was not part of the study’s weight loss program. Weight cycling was defined as one or more episodes of weight loss in the previous two years, followed by weight recovery of 5% or more of the baseline weight before weight loss. The inclusion criteria were: aged 18 to 50 years; 28 ≤ BMI < 35 kg/m^2^; ability to comply with the weight loss intervention; and a history of weight cycling. The exclusion criteria were: weight loss of more than 10% over the past year; taking medication that affects weight, glucose and lipid metabolism, blood pressure, lipids, and hormones currently or over the past six months; currently pregnant or breastfeeding women; a history of severe cardiovascular, gastrointestinal, psychiatric, and cancer diseases; with abnormal liver and kidney function; and inability to complete the study well for various reasons. The researchers examined the causes of weight loss in the participants, excluding weight loss caused by various diseases (see exclusion criteria for details) and including only weight loss caused by conscious weight loss (e.g., dieting or increased exercise) or other lifestyle intervention programs for weight loss (non-drug and surgical treatments) that were begun and completed before the participants entered the study. People with obesity and a history of weight cycling comprised 153 (53 men and 100 women) participants who completed the three-month outpatient intervention program and six-month follow-up. Pregnancy, withdrawal, missed visits, and the sudden onset of illnesses that made participation in the study impossible were all reasons for non-completion. The Human Ethics Committee of PUMC Hospital approved the study (No. ZS-3067), and all participants provided written informed consent. The study was carried out following the principles of the Helsinki Declaration. It was published in Clinical Trials (NCT05311462).

### Measurement

Participants’ height and body weight were measured using standard methods in light clothing and without shoes by a mechanical column scale (seca, China) and a bioelectrical impedance body composition analyzer (seca, China), respectively. The specialist prescribed a routine intervention program in the outpatient clinic for these participants. The diet was high-protein and energy-restricted (20–25 kcal/kg/d, of which protein was 1.5 kg/d). The exercise program included 40 min of aerobic exercise and 20 min of resistance exercise daily.

The program required six months of program adherence and daily weight recording, with weekly professional follow-up to ensure compliance. Following the completion of the intervention program, the specialist advised participants to maintain a healthy diet (following the Chinese Dietary Guidelines 2023) and check in with the online community every week. Six months of follow-up had elapsed when the article was finished, and the study had been running for nine months.

At baseline, at the end of the three-month intervention program, and after six months of follow-up, we measured participants’ height and weight. Body mass index (BMI) was calculated as BMI = weight (kg) / height (m)^2^. ΔBMI1 was the difference between BMI 1 (baseline) and BMI 2 (three months), while ΔBMI2 was the difference between BMI 1 and BMI 3 (six months). Weight loss was calculated as follow-up BMI (kg/m^2^)– baseline BMI (kg/m^2^). A questionnaire collected basic information such as name, gender, age, smoking, drinking, exercise, sleep habits, and medical histories. The questionnaire also asked all participants if they had ever tried to lose weight, how many times they had, and how much they had lost. Weight cycling was defined as having more than one weight loss episode in the previous two years and gaining more than 5% of the pre-weight loss baseline weight.

### Scales

All participants independently completed self-report questionnaires under the supervision of investigators, including sociodemographic information (e.g., age, gender, BMI, ethnicity) and the following scales, which were returned to the researchers immediately:

### Symptom-Checklist-K-9(SCL-K-9) [30]

The SCL-K-9 is a short, unidimensional version of the SCL-90-R Symptom Checklist 90 Revised (SCL-90-R) [31], a self-report questionnaire that assesses general psychiatric symptoms and psychological status and is recommended as a validated screening scale for psychological assessment by the American Society for Metabolic and Bariatric Surgery. It consists of nine SCL-90-R items (#24, #28, #31, #34, #43, #57, #58, #75, #77) and best represents (i.e., has the highest total item correlation) all of the original SCL-90-R subscales. The SCL-K-9 has been used among participants with overweight/obesity [32, 33]. Even in the absence of significant binge-eating symptoms, it has the same discriminatory validity as the SCL-90. Higher mean values indicate more severe symptoms. It suggests that the SCL-K-9 is appropriate for this study to investigate weight control correlates in participants with obesity and a history of weight cycling. SCL-K-9 was used in this study to assess psychological distress. The Cronbach’s α for this scale in the current study was 0.82 and 0.85 at baseline and three months.

### Dutch Eating Behavior Questionnaire (DEBQ) [34]

DEBQ was assessed using the Chinese version, which has high reliability and validity [35–37]. It is a self-report questionnaire used to assess adults’ eating habits. It consists of 33 items divided into three subscales: restrained eating (ten items), emotional eating (13 items), and external eating (ten items). Restrained eating is a deliberate effort to limit your food intake to control your weight. Emotional eating is eating in response to a negative emotion like stress or loneliness. External eating is eating in response to a food attraction, such as sight or smell [38]. The subscales of emotional eating and extrinsic eating were scored on a 5-point Likert scale (from 1 “never” to 5 “often”), with higher scores indicating a higher degree of emotional eating and a greater tendency to extrinsic eating. The abstinence subscales (#1, #2, #7, #8, #13, #14, #21, #22, #28, #29) were scored inversely on the 5-point Likert scale, from 1 “always” to 5 “never.” In this study, we used “reverse scoring,” meaning a higher score indicates a greater desire for the tasty food. DEBQ achieved high reliability in data collected at various time points. Cronbach’s αvalues for data collected at baseline and three months were 0.90 and 0.91, respectively.

### Power of Food Scales (PFS) [39]

PFS assesses and measures appetite and the psychological impact of living in a food-rich environment. It has three subscales representing different food proximity levels of: food available (thoughts about food generally; e.g., “I find myself thinking about food even when I’m not physically hungry”), food present (a draw toward food directly available; e.g., “If I see or smell a food I like, I get a powerful urge to eat them”), and food tasted (desire and liking of food at first taste; e.g., “When I eat delicious food I focus a lot on how good it tastes”). The scale has been used with good internal consistency among the Chinese, with a Cronbach’s α of 0.92 [40]. The Cronbach’s α for this scale in the current study was 0.90 and 0.93 at baseline and three months.

### Statistical analysis

The data was analyzed by IBM SPSS 25.0 and Amos 21.0 (IBM^®^ SPSS^®^ Amos 21.0.0). The small sample Shapiro-Wilk (S-W) test was used to determine whether the data were normally distributed. Continuous variables' means and standard deviations (SDs) were calculated and compared using the Student’s t-test or Analysis of Variance (ANOVA). The frequency and proportions of categorical variables were calculated and compared using the chi-square test. The Cronbach’s αcoefficient was used to assess internal consistency reliability, with a recommended level of 0.70 or higher indicating good internal consistency. Relationships between BMI change, psychological state (measured by SCL-K-9), eating behavior (measured by DEBQ & PFS), and levels of satiety and hunger were assessed using Pearson’s correlation coefficient.

### Path analysis

We used structural equation modeling (SEM) to test separate path analyses to determine the interactions between psychological distress and weight maintenance, eating behavior, and appetite. SEM was performed with 2,000 bootstrapped replicates using the maximum likelihood estimation method; this included eating behavior as a mediator of the relationship between psychological state and weight loss. Significant correlations (*P* < 0.05) between demographic variables and the three target variables mentioned above were added to the SEM. The path was considered significant if the 95% bias-corrected and accelerated confidence interval (95% CI) did not include zero. The following model fit indices were used as criteria for SEM: root mean square error of approximation (RMSEA; desired value < 0.06), comparative fit index (CFI; desired value ≥ 0.90), normed fit index (NFI; desired value > 0.95), goodness-of-fit index (GFI; desired value ≥ 0.90), incremental fit index (IFI; desired value ≥ 0.90), Tucker–Lewis index (TLI; desired value ≥ 0.90), and parsimony goodness-of-fit index (PGFI; desired value > 0.50). *P* < 0.01 (two-tailed) was considered statistically significant.

## Results

### Participants’ characteristics

At baseline and three months, 153 participants met the criteria for analysis. Due to pregnancy, two participants withdrew from the study at six months. Table 1 summarizes the characteristics of participants at two-time points (three-month and six-month follow-up). The participants were 34.26 (SD: 0.59) years old on average at three months. The majority were female (65.4%), married or cohabiting (50.7%), and had a bachelor’s degree or higher (85.52%). Over half of the participants took part in physical exercises (65.8%). Most participants (88.9%) never smoked and drank only occasionally (54.2%). 120 (78.4%) participants had more than two weight cycles, while 33 (21.6%) had fewer than two.

**Table 1:** Characteristics of the participants (*N* = 153)

Variable	n(%)	ΔBMI1(M ± SD)	F/t	*P*	ΔBMI2(M ± SD)	F/t	*P*
**age(mean 34.26, SD 0.59)**			1.233	0.297		1.756	0.176
20–30	56(36.6%)	3.43 ± 0.20			2.27 ± 0.32		
31–40	69(45.1%)	3.33 ± 0.21			2.84 ± 0.42		
41~	28(18.3%)	3.89 ± 0.26			3.42 ± 0.34		
**gender**			0.735	0.463		0.017	0.987
male	53(34.6%)	3.60 ± 0.24			2.76 ± 0.29		
female	100(65.4%)	3.38 ± 0.15			2.72 ± 0.32		
**marital status**			2.675	0.072		2.202	0.114
single	72(47.3%)	3.23 ± 0.20			2.30 ± 0.26		
married/cohabited	77(50.7%)	3.60 ± 0.17			3.07 ± 0.38		
divorced/separated	3(2.0%)	4.94 ± 0.76			4.67 ± 1.09		
widowed	0	0			0		
**education**			2.216	0.113		0.463	0.630
primary and below	0	0			0		
junior high school	1(0.6%)	4.6			3.77		
senior high school	3(2%)	5.27±0.83			4.18 ±1.10		
college and above	148(97.4%)	3.41±0.13			2.70 ±0.24		
**occupation**			0.738	0.620		0.760	0.603
unemployment	4(2.6%)	3.48±0.80			3.23 ±0.56		
civil servants or enterprises / institutions employees	46(30.3%)	3.17±0.26			2.18 ±0.39		
teachers	5(3.3%)	2.60±0.93			2.74 ±0.97		
soldiers	0	0			0		
retirees	2(1.3%)	3.88±0.77			2.12 ±2.06		
business and service staff	19(12.5%)	3.91±0.33			2.63 ±0.43		
workers and professional technicians	27(17.8%)	3.56±0.29			2.63 ±0.39		
others	49(32.2%)	3.57±0.23			3.37 ±0.54		
**smoke**			0.442	0.723		**3.694**	**0.013**
never	136(88.9%)	3.41±0.14			2.54 ±0.18		
below 10 pieces/day	8(5.2%)	4.02±0.65			3.06 ±0.84		
10–20 pieces/day	2(1.3%)	4.08±2.00			3.09 ±1.91		
above 20 pieces/day	0	0			0		
cessation	7(4.6%)	3.52±0.38			6.04 ±3.23		
**drink**			0.651	0.627	2.91 ±0.28	0.306	0.874
never	56(36.6%)	3.52±0.21			2.63 ±0.37		
occasionally	83(54.2%)	3.42±0.19			2.69 ±0.56		
1–2 times/week	11(7.2%)	3.37±0.34			0.22		
3–5 times/week	1(0.7%)	1.46			2.91 ±0.28		
> 5 times/week	0	0			0		
cessation	2(1.3%)	4.49±1.07			3.87 ±1.58		
**exercise**			-0.409	0.683		-1.416	0.159
no	52(34.2%)	3.45±0.19			2.33 ±0.26		
yes	100(65.8%)	3.45±0.17			2.94 ±0.32		
**sleep time**			0.953	0.388		0.020	0.981
< 6 h	27(17.6%)	3.03±0.33			2.65 ±0.47		
6–9 h	124(81%)	3.54±0.14			2.76 ±0.27		
> 9 h	2(1.3%)	3.84±0.89			2.40 ±0.04		
**weight cycling**			-0.368	0.713		0.462	0.645
< 2	33(21.6%)	3.38±0.26			2.93 ±0.77		
≥ 2	120(78.4%)	3.48±0.15			2.68 ±0.20		

Note: Data are mean ± SD for normally distributed values, median (IQR) for skewed variables, or n (%) for categorical data. Student’ t test was used to compare the mean of two groups, and analysis of variance was used to compare the mean of multiple groups. *P* < 0.05 indicates statistical significance

The mean BMI was 30.83 ± 2.16 kg/m^2^ (BMI 1) at baseline, 28.45 ± 2.27 kg/m^2^ (BMI 2) at three months, and 28.12 ± 3.54 kg/m^2^ (BMI 2) at six months. As shown in Table 1, there was no significant difference in ΔBMI1 between groups after three months. Aside from smoking (t = 3.694, *P* < 0.05), there were no differences in ΔBMI2 at six months between the variables.

Regarding the measurement scales, the overall SCL-K-9 score was moderately low, with average scores of 12.86 ± 0.36 (baseline), 11.80 ± 0.34 (three months) and 12.25 ± 4.03 (six months). The appetite and eating behavior scores during the two follow-ups were lower than the baseline. All scales and subscales had higher scores at six months than three months. Table 2 displays the results.

**Table 2:** Internal consistency and score of the SCL9, PFS, DEBQ and its three subscales

scales	baseline					3 months				6months		
	M	SD	α		M		SD	α		Μ	SD	α
**SCL9**	12.86	0.36	0.821		11.80		0.34	0.847		12.25	4.03	0.787
**PFS**	47.49	0.92	0.900		35.94		0.95	0.925		37.54	12.81	0.939
**PFS1**	17.45	0.42	0.773		13.52		0.39	0.803		14.06	5.40	0.849
**PFS2**	13.93	0.27	0.804		9.52		0.30	0.869		10.06	3.72	0.863
**PFS3**	16.11	0.35	0.819		12.91		0.35	0.848		13.30	4.64	0.862
**DEBQ**	86.33	1.42	0.903		71.62		1.56	0.910		84.74	19.68	0.916
**DEBQ1**	16.45	0.46	0.806		12.35		0.44	0.773		23.47	5.94	0.848
**DEBQ2**	35.63	1.03	0.956		30.44		1.00	0.968		31.62	12.18	0.962
**DEBQ3**	34.24	0.45	0.813		28.83		0.57	0.896		29.90	6.93	0.892
**Satiety**	72.24	1.70	/		69.08		1.44	/		69.91	16.56	/
**Hunger**	66.33	1.67	/		43.25		1.89	/		55.60	24.07	/

Note: M: mean;SD: standard deviation; α:Cronbach’s α; SCL-9: Symptom-Checklist-K-9; PFS: Power of Food Scales; PFS1: PFS of food available; PFS2: PFS of food present; PFS3: PFS of food tasted; DEBQ: Dutch Eating Behavior Questionnaire; DEBQ1: DEBQ of restrained eating; DEBQ2: DEBQ of emotional eating; DEBQ3 DEBQ of external eating

### Associations of psychosocial status, weight control levels, and modifying factors

Pearson’s correlations were used to test relationships between continuous numerical variables. The correlations between psychological distress, appetite, eating behavior, satiety, hunger, and weight change are shown in Table 3. The total scale of psychological distress was significantly positively associated with appetite (*r* = 0.164, *P* < 0.05), eating behavior (*r* = 0.324, *P* < 0.01), and hunger (*r* = 0.172, *P* < 0.05); it was negatively associated with satiety (*r* = − 0.224, *P* < 0.01). Weight changes were correlated with appetite (three months: *r* = -0.164, *P* < 0.05) and eating behavior (three months: *r* = -0.207, *P* < 0.05; six months: *r* = -0.198, *P* < 0.05). Consequently, participants with lower psychological distress, a more robust appetite, more eating behaviors, less satiety, and more hunger reported more significant weight loss.

**Table 3:** Correlations of SCL 、PFS、DEBQ and Its Three Subscales with Other Variables (*N* = 153)

Variables	SCL9	PFS	PFS1	PFS2	PFS3	DEBQ	DEBQ1	DEBQ2	DEBQ3	Satiety	Hunger	ΔBMI1	ΔBMI2
**SCL9**	1.000												
**PFS**	0.164^*^	1.000											
**PFS1**	0.177^*^	0.900^**^	1.000										
**PFS2**	0.097	0.851^**^	0.653^**^	1.000									
**PFS3**	0.143	0.878^**^	0.653^**^	0.663^**^	1.000								
**DEBQ**	0.324^**^	0.616^**^	0.581^**^	0.467^**^	0.554^**^	1.000							
**DEBQ1**	0.051	0.121	0.095	0.105	0.121	0.365^**^	1.000						
**DEBQ2**	0.362^**^	0.481^**^	0.505^**^	0.315^**^	0.410^**^	0.897^**^	0.020	1.000					
**DEBQ3**	0.139	0.711^**^	0.572^**^	0.638^**^	0.679^**^	0.721^**^	0.093	0.511^**^	1.000				
**Satiety**	-0.224^**^	0.044	0.063	0.104	-0.040	-0.080	-0.110	-0.072	0.025	1.000			
**Hunger**	0.172^*^	0.151	0.095	0.146	0.165^*^	0.089	0.125	0.033	0.076	-0.235^**^	1.000		
**ΔBMI1**	-0.017	-0.159	-0.140	-0.111	-0.164^*^	-0.118	-0.009	-0.067	-0.207^*^	0.079	-0.020	1.000	
**ΔBMI2**	-0.019	-0.109	-0.054	-0.096	-0.145	-0.108	-0.010	-0.057	-0.198^*^	0.034	0.006	0.467^**^	1.000

Note: *P < 0.05;**P < 0.01; ΔBMI1 = Body Mass Index baseline- Body Mass Index 3 months; ΔBMI2 = Body Mass Index baseline- Body Mass Index 6 months

### Mediating effect of eating behavior in the relationship between psychosocial status and weight control

**Fig. 1:**
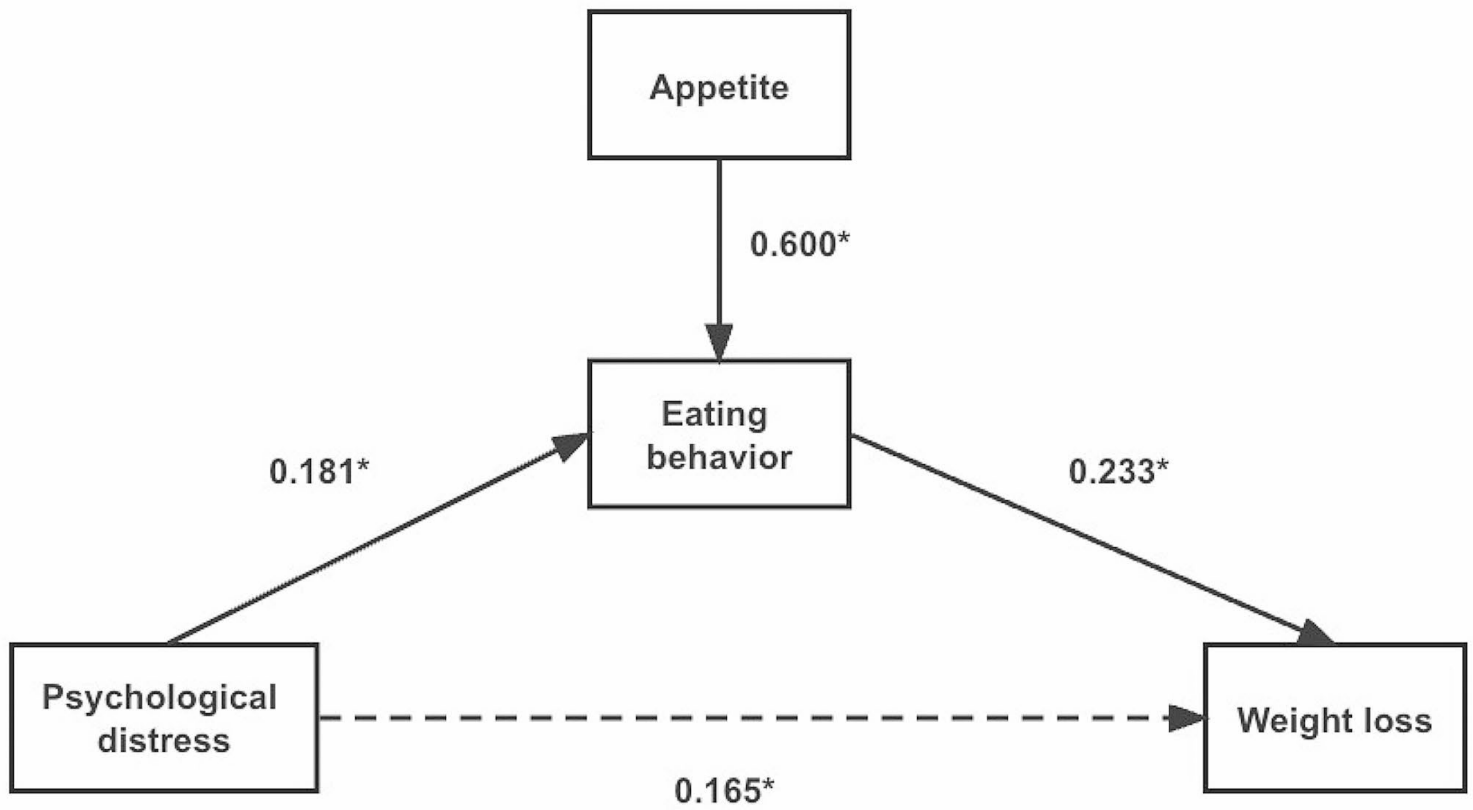
Structural model of psychological distress and ΔBMI1: Mediating role of eating behavior and appetite * *P*<0.05

**Fig. 2:**
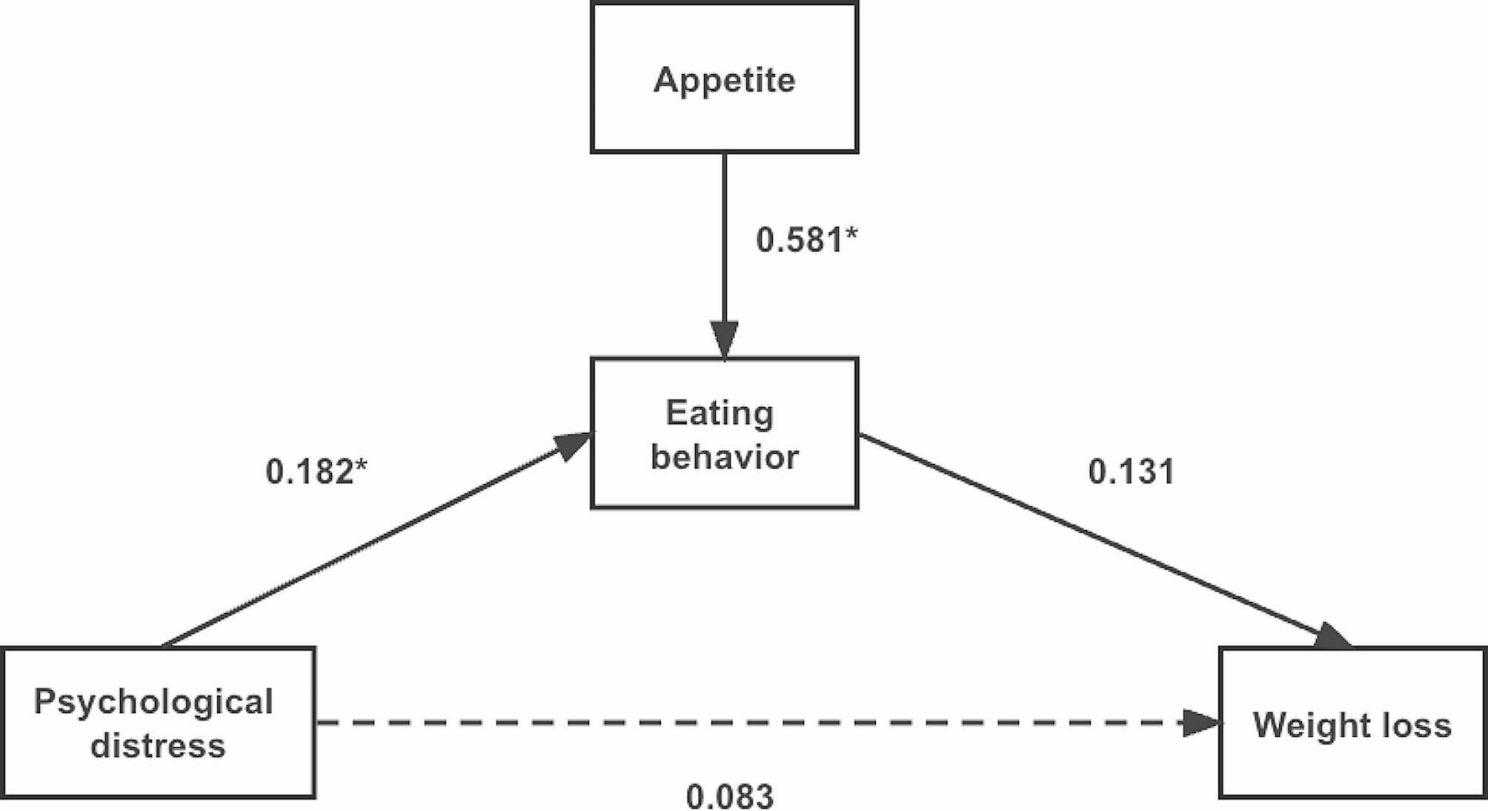
Structural model of psychological distress and ΔBMI2: Mediating role of eating behavior and appetite * *P*<0.05

Figures 1 and 2 show the variables’ standardized path coefficients in the SEM at two different time points. Tables 4 and 5 shows standardized estimates with 95% confidence intervals and *P* values at 3 and 6 month. First, after controlling for gender, age, and weight cycle times, we discovered that psychological distress’s overall effect on BMI was insignificant. It should be noted that the existence of this total association is not required for the existence of indirect associations. At three months, psychological distress was associated with more eating behaviors (β = 0.181, *P* = 0.004), and eating behavior predicted greater weight loss (β = 0.223, *P* = 0.005). The indirect effect (psychological distress → eating behavior → weight loss) was statistically significant (β = 0.181 × 0.223 = 0.040, *P* < 0.05), indicating that eating behavior mediated the association between psychological distress and weight loss. However, the indirect effect (psychological distress → eating behavior → weight loss) was statistically insignificant at six months (β = 0.182 × 0.131 = 0.023, *P* > 0.05).

**Table 4:** Standardized direct effects at 3 months

Path			Estimate(95%CI)	S.E.	C.R.			*P*
**DEBQ <---**	**SCL9**	0.181(0.055,0.310)		0.291		2.864	0.004	
**DEBQ <---**	**PFS**	0.600(0.514,0.717)		0.089		9.499	< 0.001	
**ΔBMI1 <---**	**DEBQ**	0.223(0.058,0.346)		0.007		2.830	0.005	
**ΔBMI1 <---**	**SCL9**	0.165(-0.010,0.311)		0.034		2.094	0.036	

Note: effects are significant when the upper and lower bound of the bias corrected 95% confidence intervals (CI) does not contain zero

**Table 5:** Standardized direct effects at 6 months

Path			Estimate(95%CI)	S.E.	C.R.			*P*
**DEBQ <---**	**SCL9**	0.182(0.039, 0.332)		0.063		2.903	0.004	
**DEBQ <---**	**PFS**	0.581(0.457, 0.713)		0.064		9.057	< 0.001	
**ΔBMI2 <---**	**DEBQ**	0.131(0.008, 0.274)		0.084		1.573	0.116	
**ΔBMI2 <---**	**SCL9**	0.083(-0.037, 0.204)		0.081		1.025	0.305	

Note: effects are significant when the upper and lower bound of the bias corrected 95% confidence intervals (CI) does not contain zero

Table 6 shows that the overall model of three months provided a good fit. At six months, the model fit was less than ideal. As can be seen, psychosocial distress influences weight loss at three months entirely through eating behavior, with all model fit indexes meeting the criteria except the PNFI (= 0.331, exceeding the required criteria of > 0.5). We analyzed the results six months following the three-month approach to examine the mediating effect of eating behavior in the relationship between psychosocial status and weight control in people with a history of weight cycling. We found that the results were not as good as at three months. Adding sex and age as covariates did not affect the results.

**Table 6:** Model fit indices of SEM1 and SEM2

Index name	χ2	*P*	CMIN/DF	RMSEA	CFI	NFI	GFI	TLI	IFI	PNFI
**SEM1 results**	0.715	0.699	0.358	0.001	0.999	0.992	0.998	1.044	1.014	0.331
**SEM2 results**	0.765	0.682	0.382	0.001	0.333	0.990	1.051	1.016	0.330	0.765
**Adapted standard**	-	> 0.05	< 3	< 0.05	> 0.9	> 0.9	> 0.9	> 0.9	> 0.9	> 0.5

Note: CMIN: chi-square value; DF:degree of freedom→ RMSEA: root mean square error of approximation; CFI→Comparative fit index; NFI: norm fit index; GFI: goodness-of-fit index;TLI:Tucker-Lewis Index; IFI:incremental fit index; PNFI: parsimonious normed fit index

## Discussion

The current study sought to investigate the relationship between obesity, psychological distress, and weight control. Specifically, it examined whether eating behavior mediates this relationship and whether appetite and the number of weight cycles moderate the mediating effect. We selected a population prone to weight gain and with poor weight control, i.e., a population with obesity and a history of weight cycling.

SEM was used to test associations between variables (using Spearman correlation) and to perform group comparisons by sociodemographic variables and weight loss (Student’s t-tests / ANOVA). Psychological status and appetite scores were positively interrelated and correlated with eating behavior scores and ΔBMI among participants who were weight cycling. Higher psychological distress was related to an increase in ΔBMI indirectly in our sample via eating behavior. Previous research supports the view that psychological distress is associated with weight control. However, it does not adequately explain how psychological distress affects weight control [41]. It has not been studied in depth in populations with obesity and a history of weight cycling.

Our results align with our expectations, showing that psychological distress is moderately linked to weight control measures and strongly associated with eating behavior. This suggests that psychological distress not only complicates weight control for individuals with obesity and a history of weight cycling but also heightens the risk of failing to control weight by influencing their eating behavior. Specifically, the greater the short-term improvement in psychological distress, the greater the improvement in eating behaviors, to the point of better levels of weight control six months later, within three months of these individuals attending the weight loss clinic intervention. However, the association with weight loss is insignificant after nine months, only affecting eating behavior. It suggested that participants with psychological distress issues would lose more weight if their psychological distress were addressed. Furthermore, psychological counseling is required during the early stages of weight maintenance after weight loss (three months after intervention). Long-term control (six months and beyond) may necessitate more clinical diagnosis and treatment and long-term energy intake control. However, better initial weight-loss results benefit future weight-loss confidence and results. In participants of weight cycling, this effect fully mediates; psychological distress affects BMI changes indirectly via the mediating variable (eating behavior). The structural equation modeling approach may directly investigated the relationship between psychological distress and weight control, looking for a relationship between this specific fully mediated effect. In addition, Furthermore, the indirect effect coefficient is not very large in this model, which is related to the fact that psychological distress can affect control in various ways, including mediating physiological aspects (e.g., intestinal microbiota [42]). Other factors that may have a mediating effect should be investigated further.

We hypothesized that this shift was caused by the patient’s ability to alleviate some of their psychological distress under professional physician guidance. It implies that the physician’s “to comfort always” effect played a role [43]. Previous research has found a similar relationship: emotional eating as a behavioral mechanism between depression and the development of obesity, abdominal obesity as a moderator of depression and obesity, and depression has a significant indirect effect on BMI changes [44, 45].

Factors contributing to participants’ psychological distress in those with obesity and a history of weight cycling may include depression and anxiety; lack of confidence in body image maintenance due to repeated weight loss failures; and body image anxiety influenced by social opinions [46, 47]. Outpatient physicians should pay more attention to participants with poor psychological states and provide more emotional support or multidisciplinary team interventions in conjunction with psychology clinics when seeing participants with obesity, especially those with a history of weight cycling. Future treatments such as mindfulness, meditation, and psychological counseling can be targeted [48, 49]. We recognize that income level, educational background, and employment status can significantly impact an individual’s mental health and eating habits. For instance, financial constraints or job-related stress may contribute to higher levels of psychological distress in younger adults, which may affect their eating behavior and weight maintenance. Additionally, we investigated how our participants’ youthful demographics might have specific implications. Young adults are frequently in a life transition, facing unique challenges such as career establishment, which can contribute to increased psychological distress [50, 51]. This distress can manifest in various ways, including disordered eating patterns.

This study is a cohort study of a population with obesity and weight cycling, where data is collected in stages, taking into account the short-term effects of psychological state and the effects of weight control, allowing for an assessment of causality. We also chose a specific population—a population with obesity and a history of weight cycling who are struggling with weight loss and preventing rebound [8]. The SCL-K-9, a short scale used in this study,facilitates initial screening of participants swiftly during the clinical process, helping to select suitable treatment options and identify those needing psychological multidisciplinary interventions promptly [33]. These are the advantages of this study.

We acknowledge that the current research has limitations. The data in this study come from a single-center cohort of participants with obesity and weight cycling. Because this study was conducted on young and middle-aged populations, the current study recognizes that the results may not apply to the general population. Furthermore, as with most large-scale studies, our findings depended on the reliability of each participant’s self-reported questionnaires, potentially subject to recall bias. Finally, latent variables were not introduced because the primary focus of our study was on relationships between observed variables. Although latent variables can provide a more in-depth understanding and interpretation of concepts not directly observed in the model, it was beyond the scope of our study. The lack of latent variables is acknowledged as a study limitation, and future research may consider a more thorough exploration of latent factors influencing the study model.

Weight loss was evident during the intervention (short-term), but maintaining weight is a multi-faceted physical-psychological-social effort. This study focuses on psychological distress during outpatient interventions, given that short-term psychological interventions are more achievable within treatment protocols and improving the psychological state during the follow-up period [52]. We should continue to follow up with participants in the future to obtain longer-term follow-up data, and more multi-center studies with large cohorts and longer follow-ups could be conducted.

## Conclusion

This study discovered that psychological distress was related to weight control levels in weight-cycling populations. More emphasis should be placed on psychological distress in this population. It also discovered that food behavior moderates the relationship between psychological health and weight control. Therefore, in the program of weight loss intervention, measures should be considered to reduce the psychological distress of people with a history of weight cycling to prevent the occurrence of poor weight control.

## Data Availability

All data generated or analysed during this study and its supplementary information files are included in this published article.

## Abbreviations

SEM: Structural equation model
WHO: World Health Organization
BMI: Body Mass Index
MDT: Multidisciplinary team
SCL-K-9: Symptom-Checklist-K-9
DEBQ: Dutch eating behavior questionnaire
PFS: Power of Food Scales
SDs: Standard deviations
CI: Confidence interval
RMSEA: Root mean square error of approximation
CFI: Comparative fit index
NFI: Normed fit index
GFI: Goodness-of-fit index
IFI: Incremental fit index
TLI: Tucker–Lewis index
PGFI: Parsimony goodness-of-fit index
